# Life

**Published:** 1890-03

**Authors:** W. A. Morris

**Affiliations:** President Austin District Medical Society


DANIEL'S

### Texas Medical Journal.

A Representative Organ of the Medical Profession, and an Exponent of Rational Medicine; devoted to the Organization, Advancement and Elevation of the Profession in Texas.

PUBLISHED MONTHLY.—SUBSCRIPTION $2.00 A YEAR.

Vol. V. AUSTIN, MARCH, 1890. No. 9.

### Original Articles.

CONTRIBUTED EXCLUSIVELY TO THIS JOURNAL.

*The Articles in this Department are accepted and published with the understanding that we are not responsible for, nor do we indorse the views and opinions of the writers by so doing.*

### LIFE.

[Retiring Address of W. A. Morris, M. D., President Austin District Medical Society, delivered at the Ninth Quarterly Meeting, at Austin, December 20, 1889.]

[CONTINUED FROM FEBRUARY NUMBER.]

WHEN I began the practice of medicine, blood letting was resorted to in all inflammatory affections. I do not now doubt its great value in cutting short many inflammatory diseases, and rendering them more amenable to treatment. But things changed under my own observation, and blood letting had its day. Diseases of a low type took the place of those of a more sthenic form, and venesection waned.

That blood letting was an important agent in the hands of our predecessors in the cure of disease, I cannot doubt.

We have had men of wonderful capacity to precede us. Men of observing minds and deep thought, and we cannot believe that they were not capable of judging—Dewees, Sydenham, Rush, Meigs, Southward, Smith, and a host of others.

Although I have spoken of the advancement in the different branches of knowledge in modern times, we must not therefore conclude that we are giants in intellect, for we have had grand men in the past, with as much capacity and observation as we have to-day. Step by step we are erecting the superstructure upon the foundation laid for us by them.

Our field of observation is enlarged; this generation has its part to perform, it has the experience of all the past. Grand minds have gone before us and hewn out the way; therefore let us give all praise to those men who have labored for the truth, and spent their lives in its service.

Sanitary science is of recent date. It has had a brilliant past, and has in store a more brilliant future. About a century ago, an English physician, Ambrose Parr, called the attention of the British government to the necessity of vital statistics and a system of hygiene. Acting upon his suggestion, although but imperfectly, the average of life was increased about two years..

It has only been within the last twenty years that it has taken an important hold upon the public mind. Many States have passed laws creating boards ot health, and the wisdom of such action has been fully shown by a lowering of the mortality in every state where such laws have been in operation. The death rate in many of our large cities has been reduced, in some, one-fourth, and in others even one-third. This is a great saving of life.

By a proper system of hygiene, vital statistics and preventive medicine, we may still lessen the mortality, and ultimately free our country from those frightful epidemics that frequently scourge our coasts, and desolate so many homes.

The researches of Pasteur and other co-laborers in that branch of scientific investigation, have been of great value. The value of a proper system of sanitary laws (based upon scientific principles), to mankind, cannot be overestimated, and we should not cease to agitate this important question, involving so much, till we can enlighten the public mind sufficiently to cause our lawmakers to pass such laws as will best secure the end in view.

Recent research in the physiology of the brain and nervous system has brought forth important results. Gall and Spurzheim, co-laborers and men of master minds, took great interest in this subject, and undertook to analyze the mind and to locate the different functions of the brain. They mapped out the skull by its prominences, and localized certain functions of the brain. Although most of it was erroneous, they did a great deal for science. The grand truth has now been established that there are centers in the brain that preside over the different movements of the body. The center of respiration is located in the medulla oblongata, where all the nerves, both of sensation and motion, converge and center from above and below—the great sensorium commune.

There it also a heat center, a center of taste, smell, vision, touch, language, and a center of equilibrium to co-ordinate muscular movements, and many others not named, which have recently been discovered by the interesting investigations of Ferrier, Charcot, Starr, and others.

The anatomy and physiology of the great sympathetic system which presides over organic life are now much better understood.

The cerebrum—the organ of the mind, the great emporium of thought, the throne of reason and the seat of memory, judgment and will, whose mandate controls all the voluntary movements of the body,—by virtue of it man can look within himself and see his most intimate structure and operations, and study the laws of his own being.

He is the only atom of organized life that, by the power of intellect, pierces the depths and interprets the enigma of the universe, and sees in it the infinite mind and wisdom that created all.

"Far as creation's ample range extends, the scale of sensual mental power ascends."

He can contemplate with admiration the beauty and glory and grandeur of the countless orbs moving in space, and determine the laws which govern them. He can, as it were, weigh the worlds in a balance, measure their size, ascertain their density, analyze and comprehend their elements. He has opened the book of nature, looked back upon the far distant past and read the handwriting of time, when this earth was a globe of fire moving in space; has marked out the different ages, the first beginnings of animal and vegetable life, and kept pace with its different changes as the type grew higher and higher, till at last it culminated in man—the highest.

Memory: Upon what tablets are events written? Who can explain that wonderful faculty by which the record is kept? How many millions of impressions are made upon that organized mass? The history of the nations of the earth, their languages, manners, customs, habits and literature; impressions carried from childhood to old age; incidents of past life, the recollections of which may cause emotions of joy or of sorrow, anguish and remorse, or which may mount the cheeks with a blush of shame and awaken all the emotions of the human soul. All may be there engraven, more permanently than upon steel.

Inasmuch as I do not wish to encroach too much upon the domain of metaphysics, I will only casually refer to some of the teachings of the present day.

It is taught by some that every (sensory) impression made upon the brain causes a change in its molecular composition of an unknown nature, and a new arrangement occurs, by virtue of which all those impressions are stored away for future use.

Again, it is the commonly accepted theory that in every seven or eight years all the tissues of the body undergo a change, and that part that once existed no longer exists, but that new cells have replaced the old.

If the first theory is correct, the last cannot be so, since the memory of all events would fade away as fast as new cells took the place of the old. Accepting the proposition as true, we must look upon the brain as the complicated instrument through which the mind operates and brings us into direct relation with the external world, and gives us a consciousness of our own being. Mind is most intimately associated with brain and nerve matter. The larger and more perfect the brain, all other things being equal, the greater is the power and capacity for thoughts of the individual.

It is composed largely of water, albumin, sulphur and a little phosphorous. In some respects it may be compared to a grand musical instrument, which when perfectly attuned and its cords are touched by the hand of a Mozart or a Gottschalk, pours forth the most enchanting music. The instrument is inanimate matter moved upon by mind external, both in its composition and the sweet strains of music evoked.

The brain is living matter, built up by life forces and operated upon by mind innate, associated in some mysterious manner with the organ over which it presides; hence the mind is as much involved in mystery as life, and is as inscrutable as the unveiled secrets of eternity. All human wisdom must bow down in humble submission to the mind and wisdom of Him who created the universe and governs all things according to the counsels of his own will, and created man, the wonderful being that he is.

Then, rational responsible beings made in the image of God, let us use well the faculties that God has vouchsafed to us for our well being, and press forward to the accomplishment of all that is great and good, for there is scarcely a limit to the acquisition of knowledge, or the capacity of human intellect.

Man is not only an intellectual being, but he is also endowed with moral qualities which he is in duty bound to cultivate.

No man can ever reach the measure and stature of true manhood unless he combines them both. More especially should the medical profession aspire to that exalted position which talent and morals alone can give.

Then we have reason to hope, judging from the past and relying upon the power of human mind and thought, that in the next century the discoveries that will be made in the departments of science, especially in that of scientific medicine, will far transcend our present state of knowledge, and surpass our most sanguine expectations; and that eye hath not seen, nor ear hath not heard, neither hath it entered into the heart of man to conceive the grand achievements in science which is in store for future generations.

We have to deal with organized matter controlled by living forces inherent. Therefore it is more difficult to reason from cause to effect and tell with mathematical precision how much we have, or how much vital force has accomplished.

There is no effect without a cause; nature itself is but the effect of some grand cause.

Comprehending this truth more than two thousand years ago Hypparchus began the study of the planetary bodies and their motions, and laid the foundation of astronomical science; then came Ptolemy; then Keppler; then Gallileo and Copernicus, and lastly Newton, whose power of thought penetrated the confines of the finite and grasped the mind of the Almighty and interpreted the laws that govern the universe.

Not many years ago a learned astronomer observed an aberration in the movements of the planets in a certain part of the heavens, and he reasoned and said there must be a cause and that cause a planetary body. With untiring perseverance, night after night he kept his telescope directed to that quarter, and at last it moved within his field of vision, another star added to the list already known; another triumph of human reason.

The name Man, originally, meant a thinking being; then we must think. But 'tis only by the inductive method of reasoning and a close study of the laws that govern living matter that we approximate the truth.

Duty should be your watchword. Human life is entrusted to your care; the responsibility is grave; will you be equal to the task? If so, you must labor, for knowledge is not obtained by intuition.

Says Lord Bacon: "He who seeks knowledge truly does not seek a couch whereon to rest a languid spirit, nor a terrace for a variable mind and wandering feet to walk up and down with a fair prospect; nor a fort and commanding ground for strife and contention; nor a shop for self-interest, but a rich storehouse for the glory of the Creator and the relief of man."

There is for you no bed of ease or downy pillow. No lazy, indolent man can discharge his duty. No sordid one is equal to the task.

You should visit the hut or hovel where poverty dwells, as well as the palace of the rich where no want is known.

You should go wherever suffering humanity demands your aid. No man has ever risen to distinction in any profession, without having first paid the price. It is only by constant toil and the use of the midnight lamp that the mind can be stored with rich treasures of knowledge, and when thus stored and intellect matured, he can, like the eagle when soaring aloft from the mountain top, view the plain below, and go forth prepared to make new and grander discoveries.

Permit me to say to the older men who are present to-day, your pupil age is not over. Many of our distinguished men have been, like the sturdy oak, resisting the storms of life and have worked on to old age.

Benjamin Franklin was in his eightieth year when he affixed his name to that immortal document, the Constitution of these United States.

Humboldt's "Cosmos" was finished when he was in his seventy-sixth year, and his mind continued active until his death, which occurred in his ninetieth year.

Daniel Webster died in his eightieth year. Chief Justice Marshall lived to an advanced age, and continued on the Supreme Bench a longer term of years than any other man who had occupied that position.

It has been said that the proper field of human action is humanity. Then as honorable, conscientious men we owe it to ourselves and to humanity to think and study, that we may keep up with the advancement of our profession, and although we may fail to reach the summit of fame, we will yet have that which is of priceless value,—a consciousness of having discharged our duty.

"Act well your part,
There all the honor lies."

"The steep ascent must be with toil subdued; watching and care must win the lofty prize proposed by heaven,—true bliss and real good."

"Honor rewards the good and great alone;
She spurns the indolent, the timorous and base;
Dangers and toil stand stern before her throne
And guard, so heaven decrees, the sacred place.
Who seek it must the mighty cost sustain,
And pay the price of fame—labor and care and pain."

The profession of medicine is a high and noble one. It has done more to alleviate human suffering and to promote the well-being of humanity, and has distributed more to charity than any other, and is doing more to prevent disease, lengthening life and making homes happy.

"How sweet the task to lighten human woes,
And sooth the troubled heart to soft repose.
How sweet the task the poor man's heart to cheer
And wipe from sorrow's eye the falling tear;
To clothe the naked,—to give the hungry bread,
And calm the tumult of the dying bed."

Then in conclusion let me say to you: In your intercourse with one another, be honorable, truthful and just. Our profession is too honorable and dignified to be degraded by petty jealousies or controlled by ignoble purposes. The older men in the profession should ever be ready to give a helping hand to the young who are striving for position. We were young once. The young man should not despise the counsel of his seniors. And, finally, let all be governed by the golden rule: "Do unto others as you would they should do unto you."

"Where heroes war, the foremost place I claim;
The first in danger and the first in fame."

---

"THE Snake and the Dove," by Annetta J. Halliday, the complete novel in the March Belford's, will be a genuine treat to all novel readers.

